# Family Caregivers’ Perspectives on the Potential of Drone-Based Medication Delivery in Palliative Home Care: Qualitative Focus Group Study

**DOI:** 10.2196/80320

**Published:** 2025-11-13

**Authors:** Franziska Fink, Sabrina Claudia Otto, Martin Grünthal, Anne Lehmann, Patrick Jahn

**Affiliations:** 1 Translation Region for Digitalized Healthcare, Department of Internal Medicine, Faculty of Medicine, Martin Luther University Halle-Wittenberg, Magdeburger Straße 12, Halle, 06112, Germany, 0049 345 557 6586; 2 Psychiatric Center Nordbaden, Clinic for Forensic Psychiatry and Psychotherapy, Heidelberger Str. 1, Wiesloch, 69168, Germany; 3 Pharmacy at the Bauhaus, Gropiusallee 50, Dessau-Roßlau, 06846, Germany

**Keywords:** palliative care, family caregivers, drone-based medication delivery, eHealth, technology acceptance, specialized outpatient palliative care, SOPC

## Abstract

**Background:**

Palliative care supports individuals with incurable, life-threatening illnesses, focusing on symptom management and quality of life. Access to timely care, including essential medications, is often limited, particularly in rural areas, leading to gaps in home-based care. Digital health technologies, including drone-based delivery systems, have the potential to address such logistical challenges. For these technologies to be effective, they must be adapted to the specific needs of patients and caregivers, which often differ from general health care contexts, especially in remote areas.

**Objective:**

This study aimed to identify user needs and requirements for a drone-based medication delivery system designed to supplement traditional courier services in palliative care. The study also sought to explore practical considerations for integrating drones into home-based care workflows, with the goal of informing both technical design and clinical applicability.

**Methods:**

We conducted 1 focus group (FG) and 4 semistructured individual interviews with family caregivers involved in home-based palliative care. Participants were recruited via local palliative care services in rural regions. The discussions focused on experiences with palliative care, medication logistics, needs, expectations, and concerns regarding drone-assisted delivery. Interviews and the FG were audio-recorded, transcribed, and analyzed using structured qualitative content analysis.

**Results:**

A total of 10 caregivers participated (mean age 68.6, SD 12.3 years) in this study. Six participants took part in the FG and 4 participants were part of individually conducted interviews. Caregivers frequently reported long travel distances to obtain medications, sometimes leaving patients unattended and noting medication shortages, especially in the afternoons and on weekends. Participants highlighted the potential of drones to supplement existing courier services during periods of high demand. At the same time, caregivers expressed concerns about limited technical skills, particularly regarding mobile apps for ordering deliveries, and emphasized the need for simple, user-friendly systems.

**Conclusions:**

These findings reveal logistical gaps in palliative care medication supply and design requirements for drone-based delivery systems. There is a clear demand for faster, more reliable access to essential medication, especially during evenings and weekends. Whether drone-assisted delivery can reduce hospitalizations and support patients’ wishes to remain at home warrants further research. Specialized outpatient palliative care teams are central to eHealth adoption, providing technical guidance, emotional support, and confidence in using new technologies. Their involvement in communication and training is critical. Strengthening digital health literacy requires individual training and structural adjustments to meet the needs of older caregivers and those with limited digital affinity. Concerns about losing personal interaction remain significant; digital tools should complement, not replace, face-to-face care. Timely medication access could reduce caregiver stress and improve quality of life by supporting symptom control at home. To foster acceptance, caregivers need hands-on experience. Future studies should test drone delivery in real-world settings, addressing technological, psychological, and social factors.

## Introduction

Over the past decades, most developed countries have seen declining mortality rates [1,2]. This increase in life expectancy is accompanied by a rise in chronic and incurable diseases, especially in old age, such as cardiovascular conditions, cancer, and diabetes. The end of life is often marked by enduring and heavy symptom burdens, prompting the development of specialized palliative care to support patients and families in leading a self-determined life and maintaining quality of life [3,4].

Most palliative patients prefer to remain in their familiar home environment. While 76% wish to spend their final days at home, only about 20% actually do, with nearly half dying unplanned in hospital settings [5]. The care setting significantly impacts quality of life; patients at home generally experience better outcomes than those in institutional settings [6].

As the number of terminally ill people increases, home care becomes more reliant on family caregivers. The level of support required varies depending on the illness [7]. This puts a tremendous amount of pressure on family caregivers, as they are often unable to cope with the stressful situation and may feel helpless in the face of the situation [3,8]. In this regard, it was indicated that the pattern of enduring in the social, psychological, physical, and spiritual domains at certain time points (diagnosis, return to home, recurrence, and terminal stage) is similar between patients and their caregivers, which is mainly influenced by the nature of the disease and the availability of professional care [3,9,10]. This implies that the needs of patients and caregivers should be addressed in parallel, as these key time points are identical for patients and caregivers [3,9].

Palliative care aims primarily at minimizing pain to preserve patients’ dignity and life quality, which also positively affects caregivers’ well-being [11,12]. Fast and flexible symptom management is crucial and often requires rapid medication adjustments by caregivers or specialized outpatient palliative care (SOPC) teams [13]. The inability to respond quickly and adjust necessary medication can jeopardize home care and lead to unwanted hospital admissions and a deterioration in the patient’s and caregivers’ quality of life. In other words, it is crucial to ensure rapid and direct access to essential medicines that improve quality of life, especially in rural areas.

In this context, drones may offer value by significantly improving delivery response times in health services [14]. Drones are increasingly applied in health care and medicine, particularly in areas such as public health, disaster response, telemedicine, and medical logistics [15]. In the context of medical transport, drones are used to deliver medications (eg, vaccines, pharmaceuticals), blood products, organs, defibrillators, and other essential medical equipment [15-20]. These medical drones offer substantial advantages for health care, including faster emergency response times, improved access to medical services in remote or hard-to-reach regions, and enhanced clinical outcomes, such as increased survival rates [14,21-23]. Compared with helicopters, drones offer a more cost-effective solution for transporting medical supplies. Their utility is especially pronounced in regions with challenging terrains such as mountains, deserts, or forests, in areas with limited road access, over long distances, or in locations impacted by large-scale natural disasters [18,19,24]. Overall, drones hold significant promise for improving health care delivery in such contexts [18]. Despite their considerable potential in health care, the adoption and effectiveness of drones are influenced by technological, organizational, and environmental factors (TOE barriers) [14,25,26]. In Germany and many other countries, structural and regulatory conditions, such as undefined legal frameworks and operational guidelines, currently limit the use of drones to research projects and prototype testing [14,27]. Most existing projects on medical drones have been conducted in Africa, where regulatory conditions are comparatively more flexible than in Europe or North America [27,28]. However, findings from African contexts cannot be directly generalized to European settings [29]. Among the factors affecting drone usage, usability, and design consistently emerge as the most critical. In addition, users’ limited skills, negative perceptions of the technology, and the relative immaturity of drone systems pose significant barriers to broader adoption [14,25,26].

Despite increasing interest in drone-based medication delivery, robust evidence on its impact in real-world health care logistics remains limited. Most existing studies have focused on single scenarios [20,21,23,30-32] or relied on simulations [33,34], and thus have not been conducted in real-world settings. To date, 1 study covered the entire development process, from the initial app prototypes to the complete ordering and delivery workflow using an ordering app and drone over densely populated areas in Germany [35]. In particular, to our knowledge, no study has investigated drone-based medication delivery in the real-world context of palliative care, a setting characterized by complex symptom management, urgent medication needs, and a high reliance on family caregivers. Consequently, it remains unclear whether drones could meaningfully improve medication logistics, support care networks, or enhance symptom management and quality of life in outpatient or inpatient palliative care. To address these gaps, the present study is embedded within the PalliDrone project, which aims to co-create and evaluate a drone-based medication delivery system for palliative care [36,37]. The PalliDrone project comprises multiple intervention components (T0–T3), encompassing medical and nursing strategies. It will assess the usefulness, effectiveness, and benefits of drone delivery compared to conventional courier services, treating it as a complex health intervention [38]. In this context, the present study focuses on identifying user needs, potential benefits, and practical requirements for integration into existing palliative care processes. Specifically, the research objectives are:

1. To explore the needs, expectations, and concerns of family caregivers regarding drone-based medication delivery in palliative care.
2. To assess how drone delivery could alleviate burdens on caregivers and support timely access to essential medications.
3. To identify practical and organizational factors that may facilitate or hinder the implementation of drone-based medication logistics in real-world palliative care settings.

In this study, we aim to explore the practical requirements, opportunities, and challenges associated with drone-based medication delivery in palliative care for family caregivers. Specifically, the study seeks to inform both the technical readiness of a user-centered drone delivery system and its potential integration into clinical workflows. By assessing user needs, expectations, and concerns, the study provides insights that are relevant for the development of the drone system itself as well as for its safe, effective, and feasible use in routine palliative care practice. This dual focus ensures that the intervention addresses both technological feasibility and clinical applicability.

## Methods

### Overview

This study adheres to the COREQ (Consolidated Criteria for Reporting Qualitative Research) to enhance transparency, credibility, and completeness in qualitative reporting. A completed COREQ checklist is provided in Checklist 1. The COREQ checklist was used as a guiding framework during the preparation and reporting stages. Before data collection, the checklist was instrumental in informing the study design, ensuring that all pertinent aspects—including the composition and reflexivity of the research team, participant selection strategy, and detailed data collection procedures—were meticulously planned and documented in advance. During the process of manuscript development, the COREQ items were systematically reviewed to verify that each relevant element was transparently reported in the Methods and Results sections. This process ensured completeness and facilitated reproducibility.

### Setting and Sampling

The focus group (FG) and interviews included family caregivers selected through purposive sampling. The sampling was used, with participants being purposively recruited to ensure a diverse range of experiences relevant to the study’s objective. The selection of participants was conducted by the SOPC teams of the “Anhaltische Hospiz- und Palliativgesellschaft” in Dessau-Roßlau, Germany, and the “Leipziger Palliativgesellschaft” in Torgau, Germany. Participants were approached by telephone or via email. The SOPC teams identified potential participants who met the inclusion criterion of having provided care for a palliative patient within the past 6 months in rural regions. Within this sample, variation in relationship to the patient (spouse and other relative) and gender was sought to capture a range of perspectives. Although the data collection process did not entail the application of formal stratification criteria, such as age, caregiving burden, or technology literacy, the SOPC teams were encouraged to extend invitations to individuals with a range of backgrounds, thereby fostering heterogeneity in the ensuing discussions. Group effects were minimized, and no strict exclusion criteria were applied, reflecting the open, cocreative nature of the development process. There was no relationship to participants before the study.

### Materials

A German-language FG and interview guide was developed through a systematic, multistage process informed by qualitative research principles rooted in a constructivist paradigm by AL and FF [30,39]. This method was chosen due to existing theoretical assumptions, with the aim of exploring a wide range of perspectives rather than reaching consensus. The guide focused on eliciting argumentative patterns, that is, the reasoning and justification underlying participants’ views, rather than collecting biographical narratives [40]. The stepwise procedure (collecting, checking, sorting, and subsuming) ensured systematic coverage of relevant themes while maintaining openness to emergent issues [39]. First, potentially relevant questions were collected without concern for their final usability (collecting). Next, the questions were evaluated against qualitative guideline criteria (checking). Then, the content was sorted into thematic areas (sorting). Finally, these were subsumed into question blocks, each beginning with an open introductory question. The sequence of these blocks was also determined (subsuming). Critical review and feedback were provided by PJ, MG, and members of the PalliDrone project team (peer group). Feedback aimed to assess the clarity and relevance of items. No fixed instructions were given, and the guide was refined after each feedback round.

In terms of content, the guide development was informed by established models of technology acceptance, particularly the technology acceptance model (TAM 1‐3) [41-43]. TAM proposes that technology adoption is primarily driven by perceived usefulness and perceived ease of use, complemented in later extensions by factors such as social, cognitive, and psychological factors. These factors collectively shape the intention to use. The Technology Usage Inventory expands upon the traditional technology acceptance factors of the TAM, such as perceived usability, usefulness, immersion, and accessibility (technology-specific), by incorporating key psychological constructs, including interest, curiosity, technology-related anxiety, and skepticism [44,45]. The guide built on these theoretical foundations and on instruments from previous research on cocreative development of drone-assisted delivery and mobile app [30]. Thus, the final guide includes four categories: (1) processes and problem situations in palliative care (is-state), (2) specification of the process flow and communication (usability), (3) possibilities for realizing technical integration (usefulness), and (4) concerns regarding the implementation and integration (concerns). The guide began with a narrative stimulus (description) to initiate the conversation, followed by open-ended thematic question blocks. These were built on previous responses (immanent) and introduced new themes (exmanent). Transitions between blocks were deliberately soft to encourage storytelling. Evaluative questions were asked at the end. The complete FG and interview guide is available in Multimedia Appendix 1.

### Data Collection

All FG and interview discussions were conducted in German. The FG was conducted at “Leipziger Palliativgesellschaft” in Torgau, Germany. With the help of the “Anhalt-Hospiz Dessau”, interviews were conducted at the homes of caregivers. Two moderators (M) guided both formats: M1 (AL, female, research associate, experienced in mixed methods) actively led the sessions using an inductive questioning approach (starting with immanent before exmanent questions), while M2 (FF, female, postdoc, experienced in FG and interviews) remained more passive and managed the input setting. M1 moderated the discussion, asked questions, and encouraged participants to elaborate, drawing out a range of views. M2 documented interactions, atmosphere, and key statements through an ad-hoc protocol, which was later made available for participants to verify the accuracy of their contributions. Before the data collection, M1 received interview training from M2. Both moderators were familiar with the subject matter and guided their actions using the principle of “methodological understanding of others” [46].

At the beginning of each session, moderators introduced themselves and explained the procedure. A detailed overview of drone-based medication delivery was presented, followed by an open discussion about possible applications in palliative care. Participants reflected on urgency, expected relief for caregivers, and situations where traditional courier delivery would suffice.

Sessions were audio-recorded. Data validation was ensured through member checking. Participants received summaries of their respective FG discussions or interviews and were invited to provide feedback or corrections. This process helped to confirm the accuracy and credibility of the data while respecting participants’ time and availability.

Participants were informed in writing and verbally about the recording and their right to withdraw consent at any time.

The FG lasted about 90 minutes; interviews lasted around 30 minutes, depending on the situation. In interviews, 1 member of the SOPC team was present, and in the FG, 2 members were present. This was done deliberately to provide emotional support for participants, given that recruitment targeted caregivers within 6 months of bereavement, which could evoke distress.

### Data Analyses

All collected statements during the FG and interviews were repeated and discussed with participants. All participants confirmed for correctness. The participants’ responses were transcribed and coded by 1 researcher in German (AL). Transcripts were not returned to participants. A simple transcription system was used, that is, only manifest content was analyzed [47,48]. The data analysis was conducted according to the content analysis method from Elo and Kyngäs [47] and was coded using a deductive approach moving from general to specific [49]. All data were coded with the MAXQDA 2022 program (VERBI Software GmbH). The unit of analysis (event) was defined as words and as one or several sentences that contain one theme of the guideline-based questions [50]. Thus, aspects that would fit the matrix were chosen from the data. The categories were formed along the guideline-based question complexes [30,44] (see Table 1).

To validate the concept, a second researcher (FF), familiar with the analyses, independently coded 20% of the transcripts to calculate the interrater reliability. In case of discrepancies, discussions were held between the 2 researchers until a consensus was reached. Cohen Kappa was computed for all variables [51]. The interrater reliability was κ=0.71 (*P*<.001) with a substantial agreement [51,52].

For reporting purposes, illustrative quotes and examples were translated into English. Translations were iteratively cross-checked by FF and AL of the research team to ensure that the original meaning, tone, and context were preserved.

**Table 1.** Structured analysis matrix showing the categorization of caregiver statements collected during focus groups and interviews. The study explored perspectives on drone-based medication delivery among family caregivers of recently deceased palliative patients (within 6 months) in rural areas in Germany, in 2024.

No.	Category	Definition	Example
1	Is-state	Describes the initial situation and current state of problems and work procedures in palliative care as well as the competence in handling medical apps and drones	—^a^
1.1	Work procedure	Describes the current work flow in palliative care	“We always plan for a week and adjust it every day, depending on what we need.”
1.2	Problems	Describes current problems in palliative care that justify the usefulness of the technology	“So we have to speed things up so that we can do all this.”
1.3	Competence	Describes the current level of knowledge and skills in handling medical apps and drones	“Well, I have no experience. Sometimes you see one flying and it’s a bit easy.”
2	Usability	Describes the necessities or conditions for the comprehensibility, application, and usability of the technology	—
2.1	Realization options	Describes different implementation options for the drone, app, and the general process	“It would really be more interesting for rural areas, where there is a bit more space and people have their yard.”
2.2	Communication	Describes the necessities or conditions for the communication of the technology	"I could imagine that. But then it has to be quick. Because when I call, I’m on a house call and a normal patient, I want to prescribe an antibiotic. It’s the weekend, Saturday if you like, I need it on duty today if you like and then I call. Then it’s really a matter of a minute and I don’t stay in this house call forever. And then I think it has to happen within five minutes.”
2.3	Drone process	Describes the necessities or conditions/characteristics of the process of technology	“If you are logged in, you will see all patients. When I log in, I also see all patients.”
2.4	Ordering	Describes the necessities or conditions for the ordering of medication	“But that’s basically just triggering the prescription. I could also call. I think in this case it’s quite practical via this app. Triggering the prescription.”
2.5	Delivery	Describes the necessities or conditions for the transfer of medication	“For example: It should be dropped off right here on the meadow, or somewhere up ahead. And when the person of SOPC team, triggers the order, they can decide: Delivery here or to the customer. And the caregiver can only decide: Delivery to my home.”
3	Usefulness	Describes the advantages, the benefits of the technology	"We’ve had it once or twice now, we had a ward round with the doctor. She wanted to inject a medication, but we didn’t have it there. It would be good to say, I need this now. It’ll be there in 10 min.”
4	Concerns	Describes the dangers, the risk, the complications, the disadvantages, the difficulties that the technology brings with it	"I think there are problems with the fact that the patients we look after can’t do that at all. [...] So I say, if you’re there as a nurse yourself and you have the app on your duty phone, it may all work. You can use it, but the patients we look after, I’d say 70, 80 percent of them, can’t do that. And they’re not mobile either, they’re immobile, they can’t even go out the door. I think that’s actually the big problem.”

a Not available.

### Ethical Considerations

The PalliDrone project was reviewed and approved by the Ethics Committee of Martin-Luther-University Halle-Wittenberg (case number 2024‐038, date of approval April 18, 2024) for the conduct of the PalliDrone study. All participants gave their informed written consent before the FG and semistructured interviews and were informed about the procedure, the general aim, and the reasons for the discussion or interview. Participants were informed of their right to withdraw at any time without any consequences. For participants who were approached for qualitative interviews following recent bereavement, special care was taken to ensure they had the opportunity to ask questions and receive adequate explanations before consenting. All data were anonymized before analysis. All personal identifiers (eg, names and locations) were removed during transcription. Transcripts and coded data were stored on secure, password-protected servers accessible only to the research team. Participants did not receive financial compensation for their participation but were offered reimbursement of travel expenses where applicable. No identifiable images or other identifying information about participants are presented in this manuscript or in the supplementary materials.

## Results

### Characteristics of Caregivers

Between June and July 2024, we collected data from 10 participants. Six participants took part in the FG, and 4 participants were part of individually conducted interviews. No one dropped out during the discussions or interviews. All caregivers (n=10; mean age 68.6, SD 12.3) lacked knowledge and skills in using drones, and only 30% (3/10) were familiar with using medication apps. Of the 10 participants, 30% (n=3) care for their relatives while they are still working. Additional findings are reported in Table 2.

**Table 2.** Demographic characteristics of study participants, including age, gender, technology competence, and education (N=10). Participants were family caregivers of recently deceased palliative patients (within 6 months) in rural areas in Germany.

Characteristic	Value
Age, mean (SD)	68.60 (12.29)
Age range (years)	49‐84
Gender, n	
Female	8
Male	2
Drone competence, n	0
Medication app competence, n	3
Professional active, n	3
Secondary school, n	6
High school, n	3
Profession degree, n	5
University degree, n	5

### Qualitative Content Analyses

#### Overview

##### Is-State

With 80‐81 utterances, talking about their daily lives and problems was the most reported topic during the interviews and FG. However, all participants reported that they worked well with the SOPC.

*[…] I’ve already called at night when he gets his pain attacks again, […] it’s wonderful, they [SOPC] were always available.*

Some of the participants reported that they had little or no technical experience (competence), did not own a tablet or smartphone, and therefore could not imagine ordering medication via app and drone. Technical difficulties or poor network coverage in rural areas are barriers to the use of drone-based medication delivery (problems). In particular, those who reported no problems with medication delivery said they did not see the point of having medication delivered by drone. While some of the caregivers stated that the medication delivery with the courier service has been good so far, most of the participants reported problems with long errands for medication and transportation tickets. One of the biggest difficulties is leaving their relatives alone for too long:

*You come home and he’s lying down again because he tried to get up and he couldn’t. But those are situations where you’re really pushing yourself to the limit. I could see [the drone] really taking the pressure off.*

They mentioned that home delivery may be a relief in any case.

Another major problem mentioned was delivery bottlenecks during evenings and weekends. One woman described the problem this way:

*I also took over the final care of my uncle. He had lung cancer. And he was taken out of the hospital without medication. He was screaming in pain. I had an aunt who was completely overwhelmed. […] I got in the car and drove to the next town. Friday night. Pharmacies closed. No pharmacy available. […] We just didn’t have the medicine. […] in the morning we had the same problem. It’s a small town, but the pharmacy had been closed for three months because the owner was sick. So we had to go back to the next bigger town. Same problem. Who should I send? My aunt couldn’t have made it. […] I was really lucky that the doctor came by chance, his family doctor, and they knew each other privately. She said I’ll stay here until you get back and I drove myself. But that’s where the problems start. If I had a drone, a lot of things would have been easier for me.*

In addition, many caregivers have little family support, are often older adults, are no longer able to drive, or have to travel long distances, making it difficult to provide care and obtain medications. The lack of time available to general practitioners was also mentioned as a problem.

Another mentioned problem was the e-prescription. A key issue with e-prescriptions is delayed availability; pharmacies often cannot access all medications immediately after the chip card is inserted, as updates may take additional time.

##### Usability

Participants emphasized that the delivery process should be clear and as simple as possible. Since caregivers often need more than just medication—such as transport tickets—they suggested drones could also deliver important documents. Generally, they found it difficult to imagine ordering medication via an app and drone but appreciated the idea of centralizing all information: “because then you only have one point of contact.*”* Most participants could only envision drone delivery being useful in rural areas.

In terms of communication, participants stressed the importance of direct contact with SOPC services, especially for consultations:

*And there are also side effects from these medications. That’s the case when you have palliative care and questions quickly arise.*

They would be more willing to use an app if it enabled phone communication with general practitioners, SOPC teams, and pharmacies. A chat function was dismissed as too impersonal and lacking advisory value. One participant with a caregiver who spoke limited German suggested the app should support multiple languages.

Some participants preferred that the SOPC team initiate medication orders through the app, with individual orders placed only if necessary. They also valued having a choice between courier and drone delivery.

Regarding logistics, caregivers suggested that *“*the nursing service should be involved” and that medication be delivered directly to the SOPC service. While most found it hard to visualize the drone process, “find it hard to imagine how such a drone would work,” they could imagine choosing half-hour delivery time slots and having “an alternative” between courier or drone.

##### Usefulness

Although usefulness was in sum the category with the lowest statements, it was the category with the most agreement that drones would be useful in “emergency situation where they’re panicking or something,” in rural areas, “over the weekend,” or in situations where the relatives have to be left alone:

*My husband was very ill at the time, he always said when I had to go away […]: Don’t leave me alone. There was no one there […]. It’s all such a problem […] so with the drone, that’s a good thing.*

##### Concerns

Participants encounter a range of challenges when delivering medications via drone. These include adverse weather conditions, inadequate network infrastructure, dead spots in remote areas, malfunctioning technology, and users who are unable to operate the technology. In addition, data protection concerns and escalating costs for users further compound the challenges. Notably, the primary concern among participants pertains to technical issues with the drone, such as its ability to reach its destination and its reliability. There is also a fear that “the drone may crash.”

The participants expressed concerns that the introduction of drone-based medication delivery systems might result in a diminution of human interaction with pharmaceutical services:

*I can well imagine that if someone is alone at home and then only has a telephone, they no longer have any relationship with other people. That’s why I think it’s very important for me, […] that there is [...] personal contact, with the doctor or, as is the case with palliative care, that human closeness is not lost.*

This is accompanied by *“*the concern that if you take advantage of this, you might get one less home visit.”

Another concern raised was the logistics of medication delivery by drone if “people […] live in a new building, three floors up, are single, old, over 80 and even older, then the drone comes and drops the medicine in front of the door, downstairs, who’s going to fetch it?*”*

This concern was further compounded by the possibility that the delivery would be made to a lower floor, necessitating the retrieval of the medicine by someone else, like children or pets. The participants expressed uncertainty regarding the procedural mechanisms involved in the administration of medication delivered by drone. Specifically, there was a lack of clarity concerning the necessity of signature authentication in such circumstances, given the assumption that the delivery would be formalized through the usage of a signature.

## Discussion

### Principal Findings

This study aimed to explore the practical requirements, opportunities, and challenges associated with drone-based medication delivery in palliative care for family caregivers by addressing their needs, relief, and requirements. FG and interview results revealed that family caregivers face challenges in assessing the usefulness of drone delivery, largely due to unfamiliarity with such technologies and generally low eHealth literacy [53]. Caregivers highlighted frequent delays in medication access, especially during evenings and weekends, which often led to emergency interventions and increased stress. They expressed a need for reliable and fast delivery solutions that operate beyond regular hours, while emphasizing the importance of maintaining human contact and trusted communication channels, particularly with SOPC teams.

### Needs, Expectations, and Concerns of Family Caregivers Regarding Drone-Based Medication Delivery in Palliative Care

This study revealed that family caregivers face persistent challenges in accessing medications in a timely manner, particularly during evenings and weekends. These delivery gaps often result in emergency interventions or unplanned hospital admissions, increasing stress and contradicting patients’ wishes to remain at home [5,6]. Such problems are especially acute during early stages of palliative care, before SOPC teams are established [3,8-10]. This highlights the need for a reliable, fast delivery system that operates even during off-hours, where conventional courier services may fail. Studies in rural medicine and emergency supply delivery have demonstrated drones’ ability to overcome logistical and geographic barriers and reduce delivery times, especially in areas with limited infrastructure [15-24,32]. For example, the usage of drones for the delivery of defibrillators has led to a notable enhancement in accessibility to essential lifesaving equipment [21-23,32]. Consistent with the findings of our study, these investigations underscore the value of drones as a useful addition to existing logistics, rather than a replacement. They demonstrate the potential of drones to facilitate access to critical medical supplies through faster delivery [54]. However, there is a paucity of studies that have used drones within the framework of a living lab design to substantiate their effectiveness and practicality within a particular context. Moreover, palliative care differs from emergency contexts in its ongoing, sensitive care relationships where continuity and human interaction are paramount.

However, the family caregivers also highlighted a limited familiarity with drones and generally low eHealth literacy, making it difficult to assess the usefulness of the technology. The technological gap makes it difficult to assess what caregivers truly need in this context. This gap necessitates empowering both individuals through skill development and adapting social and technical infrastructures accordingly, because digital health literacy is an interplay between user competence, system readiness, and their interaction [55]. At the same time, the likelihood of requiring palliative care increases with age, raising concerns about a mismatch between technical competence and care needs. Although the most common palliative care services are symptom management (88%) and psychological support (81%), which often require face-to-face contact, eHealth can complement traditional support to some extent [56]. However, concerns regarding a potential loss of trusted human contact, particularly with SOPC teams, privacy risks, and the possibility that digital ordering systems could exclude those with limited technical competence were faced in this context. These concerns align with previous studies reporting that palliative care users value face-to-face relationships and clear communication as part of care delivery [53,57]. This indicates that the loss of human connection is a key concern, mirroring previous research [31,53,58]. Given disparities in access to palliative care globally, alternative delivery models that help patients and families communicate needs are essential [59]. This is particularly important for family caregivers, who frequently report feelings of helplessness in managing complex care situations [8,60].

### Drone-Assisted Delivery as a Relief for Family Caregivers

Despite these concerns, caregivers acknowledged the potential benefits of drones, especially for logistical relief and emergency access. Participants described how delivery delays often required them to travel long distances to pharmacies, leaving patients unattended. These situations were particularly stressful for older adult caregivers, many of whom had limited mobility or transport options [61,62]. A reliable drone delivery system was perceived as potentially reducing such logistical burdens by shortening delivery times and enabling rapid response during urgent situations, especially in rural or poorly connected areas [15-24,32]. Although most participants had no previous experience with drones, they acknowledged that faster access to medication could improve symptom control and help maintain patient comfort, which in turn might reduce caregivers’ stress levels. Previous studies have demonstrated strong links between patient well-being and caregiver quality of life [3,7,10,62,63]. Managing pain effectively is a priority in palliative care, and fears related to pain, particularly fear of patients dying in pain, can exacerbate caregiver distress [63]. Faster access to medications via drones could reduce these fears and feelings of helplessness [21-23,32]. However, these anticipated benefits remain hypothetical and require further empirical testing in real-world palliative care contexts.

### Practical and Organizational Factors for Implementation of Drone-Assisted Delivery

Several conditions emerged as critical for successful implementation. Integration into established SOPC workflows was considered essential to maintain trusted communication channels and ensure that drones complemented rather than replaced conventional courier services [53,57]. Strengthening collaboration with SOPC teams is crucial, as caregivers view these professionals as trusted partners for both emotional and technical support. Participants emphasized that the ordering process should be as clear and simple as possible, ideally with the SOPC team initiating medication orders and individual orders placed only when necessary. They valued having a single point of contact for all medication-related information (eg, within the app) and suggested that drones could also transport essential documents, such as transport tickets. Direct communication options, particularly via telephone, with SOPC teams, general practitioners, and pharmacies were seen as indispensable, while chat functions were dismissed as impersonal and lacking advisory value. Direct communication was also the most important aspect of the cocreative process for developing a delivery app for drone-assisted systems [31]. However, in the present study, participants were, on average, 18 years older (mean age 68.6,SD 12.3) and preferred not to communicate with the SOPC via app or chat. Moreover, multilingual app interfaces were recommended to accommodate caregivers with limited German proficiency.

In terms of logistics, caregivers expressed a preference for having the option to choose between courier and drone delivery, with flexible time slots (eg, 30-minute windows) for urgent deliveries. Involving nursing services and enabling direct delivery to SOPC facilities were seen as beneficial. While many participants found it difficult to imagine the operational aspects of drones, they recognized their potential for rural areas with limited infrastructure.

Environmental and technical challenges, such as weather conditions, potential mechanical failure, and flight range limitations, were viewed as possible constraints. Data protection, noise disturbance, and regulatory and liability issues were additional concerns that would need to be resolved before deployment [26,31]. Moreover, unequal access to necessary digital tools could exacerbate disparities among caregivers and patients from different socio-economic or geographic backgrounds [31]. These findings highlight that technological readiness alone is insufficient; social, infrastructural, and regulatory readiness must also be addressed before implementation [25,55].

### Study Limitations

Although the sample reflects typical age and gender distributions, it is not representative due to the small number of participants. The recruitment process, conducted by SOPC staff, may have introduced a positive selection bias. Caregivers with an established trust relationship with the SOPC teams may have been more inclined to participate and potentially more open to innovative care concepts, including drone delivery. This could have led to an overrepresentation of participants who were generally supportive of new interventions. We also note that such participants may have been more motivated to engage in research activities, which could have influenced the generally positive reception of the concept. While drones in medical delivery were largely unknown to all participants, reducing the likelihood that only technology enthusiasts were included, clear rejections of drone use were rare. However, limited knowledge restricted the depth of the discussion. Nevertheless, data saturation can be assumed, as previous studies have also shown low awareness of drones in health care, likely due to the novelty of the technology and lack of discourse [31]. Despite this, the discussions revealed a clear need for drone delivery, particularly through the detailed accounts of caregivers’ challenges. This supports the relevance of the topic, even with limited previous knowledge. Another limitation concerns the presence of SOPC team members during data collection, one in the interviews and 2 in the focus group. Their involvement was intended to provide emotional support for participants, who were within 6 months of bereavement. However, the SPOC involvement may have introduced social desirability bias, as SOPC staff were also involved in recruitment. Participants might have felt inclined to provide answers they believed aligned with SOPC expectations. To minimize this risk, SOPC staff did not actively contribute to the discussions, and all facilitation was conducted by the research team. However, the possibility that their presence influenced participants’ responses cannot be fully excluded. All FGs were conducted in German; to preserve meaning, translations were cross-checked. Minor linguistic shifts were adjusted through a process of iterative translation.

### Clinical Implications

This study reveals several clinical implications. First, integrating eHealth can help ensure timely medication supply during critical care gaps, such as evenings and weekends. Drone-based delivery could reduce unnecessary hospitalizations and support patients’ preference to remain at home. Second, SOPC teams play a central role in facilitating eHealth use. Caregivers view them as essential for technical guidance, emotional support, and confidence in using new technologies. Their involvement in training and communication is therefore crucial. Third, strengthening digital health literacy should be a structural task. The study shows that a lack of eHealth literacy is a key obstacle to accepting and effectively using drone-based health care solutions. Therefore, empowerment measures should focus not only on individual training but also on structural adjustments that better adapt technological offerings to the abilities and needs of target groups, such as older relatives and people with little affinity for technology. Fourth, technology must not replace human connection. Concerns about losing personal interaction in palliative care remain significant. Digital tools should complement in-person care and include communication features that maintain links with SOPC teams. Fifth, improving the quality of life through faster access to medication may ease pain and reduce caregiver stress. Drone delivery may help close critical supply gaps, a potential that future research should explore further. Ultimately, to foster acceptance, caregivers need hands-on experience with drone-based services to understand their practical value.

### Conclusions

This study highlights both opportunities and challenges in implementing drone-based medication delivery in palliative care. Caregivers perceived drones as a promising solution for timely access to essential medication, particularly during critical care gaps such as evenings and weekends. It remains open for further research whether the integration of eHealth solutions like drone-assisted delivery could help reduce unnecessary hospitalizations to support patients’ preference to remain at home.

Successful adoption; however, requires more than technical feasibility. Low digital health literacy, especially among older caregivers, remains a key barrier. Effective eHealth must align with users’ abilities, offering simple interfaces and integration into trusted systems like SOPC teams, who play a central role in providing technical guidance, emotional support, and confidence in new technologies. Their involvement in both communication and training processes is therefore critical for successful adoption. Strengthening digital health literacy should be addressed not only through individual training but also via structural adjustments that better adapt technology to the needs of older caregivers and those with limited affinity for digital tools.

Improving timely medication access could also reduce caregiver stress and improve patients’ quality of life by supporting symptom control at home, which remains open for further research. Ultimately, to foster acceptance, caregivers need hands-on experience with drone-based services to understand their practical value. Future research should focus on real-world testing in living lab environments, evaluating feasibility, effectiveness, and integration into existing clinical workflows while ensuring that technological, psychological, and social needs are equally addressed.

## Supplementary material

10.2196/80320 Multimedia Appendix 1 Interview guide.

10.2196/80320 Checklist 1 COREQ checklist.

## Abbreviations

COREQ: Consolidated Criteria for Reporting Qualitative Research
FG: focus group
SOPC: specialized outpatient palliative care
TAM: technology acceptance model
