# Supplementary material for: Family Caregivers’ Perspectives on the Potential of Drone-Based Medication Delivery in Palliative Home Care: Qualitative Focus Group Study
Source: JMIR Hum Factors. 2025 Nov 13;12:e80320. doi: 10.2196/80320 (PMC12614115; doi:10.2196/80320)
Supplement: Multimedia Appendix 1 [file humanfactors-v12-e80320-s001.docx]

**Interview Guide**

Opener:

M1: “Thank you very much for taking the time to talk about drone-based medical care for palliative patients in inpatient and outpatient care. Your contribution will help us to investigate and redesign the care situation in palliative medicine. We have invited you today because you are in close contact with palliative care patients [specialized outpatient palliative care (SOPC) staff, caregiving relatives] and you are significantly involved in ensuring the care of these patients. We would like to find out your opinions on this topic today. We will audio record this conversation. We need this for the evaluation afterwards. The audio will be deleted after the evaluation. We will start by briefly introducing ourselves (M 1 and M 2 begin).”

**Table S2.** Interview guide questions assigned to category based on Fink et al. (2024).

| **No.** | **category** | **category** | **Question (expamples)** |
| --- | --- | --- | --- |
|  |  |  |  |
| 1 | is-state | work procedure | Please tell us what your working day in palliative care / your everyday life with the relative you are caring for usually looks like. Please tell us about your previous experiences with (work) procedures, as well as the organization of medication for palliative care patients / for your family member in need of care. |
|  |  |  |  |
| 2 | is-state | problems | What problems do you regularly face in palliative care and why? |
|  |  |  |  |
| 3 | is-state | knowledge and competence | Do you have knowledge and competence in handling with medical apps and drones? |
|  |  |  |  |
| 4 | usability | drone process | How do you imagine the process in the delivery of drugs? Can you imagine it well? What features needs the delivery process? How would the process be as barrier-free as possible? |
|  |  |  |  |
| 5 | usability | communication | Which forms of communication within the technology are conceivable and useful for you? |
|  |  |  |  |
| 6 | usability | ordering and handover | Should patients, family caregivers and SAPV staff be able to place and manage medication orders?  Which safety-relevant aspects are important to you in connection with the delivery of medication and why? |
|  |  |  |  |
| 7 | usability | delivery | What are the necessary conditions and functionalities of the app and drone deliveries? What is the optimal drone delivery process for you? |
|  |  |  |  |
| 8 | usefulness | usefulness | Do you see a need for additional medication logistics in the form of drone-based delivery? |
|  |  |  |  |
| 9 | concerns | skepticism and fearfulness | Do you have any other suggestions or concerns you want to let us know? |

References

Fink, Franziska; Kalter, Ivonne; Steindorff, Jenny-Victoria; Helmbold, Hans Konrad; Paulicke, Denny; Jahn, Patrick (2024): Identifying Factors of User Acceptance of a Drone-Based Medication Delivery: User-Centered Design Approach. In: *JMIR human factors* 11, e51587. DOI: 10.2196/51587.
