# Supplementary material for: Family Caregivers’ Perspectives on the Potential of Drone-Based Medication Delivery in Palliative Home Care: Qualitative Focus Group Study
Source: JMIR Hum Factors. 2025 Nov 13;12:e80320. doi: 10.2196/80320 (PMC12614115; doi:10.2196/80320)
Supplement: Checklist 1 [file humanfactors-v12-e80320-s002.pdf]

S1 Table. COREQ Checklist.

No	Item	Guide questions	Answer	Location where item is reported
**Domain 1: Research term and reflexivity**				
Personal Characteristics				
1.	Interviewer/facilitator	Which author/s conducted the interview or focus group?	M1: AL<br>M2: FF	section 2.1.3 Data Collection
2.	Credentials	What were the researcher's credentials?	M1: research associate<br>M2: PhD	section 2.1.3 Data Collection
3.	Occupation	What was their occupation at the time of the study?	M1: research associate<br>M2: postdoc	section 2.1.3 Data Collection
4.	Gender	Was the researcher male or female?	AL: female<br>FF: female	section 2.1.3 Data Collection
5.	Experience and training	What experience or training did the researcher have?	AL: experience in moxed-method studies<br>FF: experience in mixed-method studies, interviews and focus groups	section 2.1.3 Data Collection
Relationship with participants				
6.	Relationship established	Was a relationship established prior to study commencement?	no	section 2.1.1 Participants
7.	Participant knowledge of the interviewer	What did the participants know about the researcher?	aim and reasons for doing the research	section 2.1.1 Participants
8.	Interviewer characteristics	What characteristics were reported about the interviewer/facilitator?	name, researchers credentials, position in project	section 2.1.3 Data Collection
**Domain 2: study design**				
Theoretical framework				
9.	Methodological orientation and Theory	What methodological orientation was stated to underpin the study?	focus groups, interviews, content analysis	2.1.1 Participants – 2.1.4 Data Analyses
Participant selection				

10.	Sampling	How were participants selected?	purposive, snowball	2.1.1 Partici-pants
11.	Method of approach	How were participants approa-ched?	telephone, email	2.1.1 Partici-pants
12.	Sample size	How many participants were in the study?	10 participants	3.1 Char-acteris-tics of Caregiv-ers
13.	Non-participation	How many people refused to par-ticipate or dropped out? Reasons?	n/a	3.1 Char-acteris-tics of Caregiv-ers
Setting				
14.	Setting of data collection	Where was the data collected?	at "Leipziger Palliativge-sellschaft" in Torgau, Germany or at home of caregivers	2.1.3 Data Collec-tion
15.	Presence of non-partici-pants	Was anyone else present besides the participants and researchers?	no	2.1.3 Data Collec-tion
16.	Description of sample	What are the important character-istics of the sample?	gender, age, drone com-petence	3.1 Char-acteris-tics of Caregiv-ers, Ta-ble 2
Data Collection				
17.	Interview guide	Were questions, prompts, guides provided by the authors? Was it pilot tested?	Questions were provided by authors; no pilot test-ing was conducted but focus group instrument was peer-group devel-oped	2.1.2 Materials / 2.1.3 Data Collec-tion
18.	Repeat interviews	Were repeat interviews carried out? If yes, how many?	no	1 Intro-duction
19.	Audio/visual recording	Did the research use audio or vis-ual recording to collect the data?	audio recording	2.1.3 Data Collec-tion
20.	Field notes	Were field notes made during and/or after the interview or focus group?	yes	2.1.3 Data Collec-tion
21.	Duration	What was the duration of the inter-views or focus group?	focus group: 90 minutes; interviews: 30 minutes	2.1.3 Data Collec-tion
22.	Data saturation	Was data saturation discussed?		4.1 Study Limita-tions

23.	Transcripts returned	Were transcripts returned to participants for comment and/or correction?	no, but field notes were made available for participants for comments and corrections	2.1.3 Data Collection
**Domain 3: analysis and findings**				
Data analysis				
24.	Number of data coders	How many data coders coded the data?	two (FF and AL)	2.1.4 Data Analyses
25.	Description of the coding tree	Did authors provide a description of the coding tree?	yes	2.1.4 Data Analyses, Table 1
26.	Derivation of themes	Were themes identified in advance or derived from the data?	in advance	2.1.4 Data Analyses
27.	Software	What software, if applicable, was used to manage the data?	MAXQDA	2.1.4 Data Analyses
28.	Participant checking	Did participants provide feedback on the findings?	yes	2.1.4 Data Analyses
Reporting				
29.	Quotations presented	Were participant quotations presented to illustrate the themes / findings? Was each quotation identified?	yes	3.2 Qualitative content analyses
30.	Data and findings consistent	Was there consistency between the data presented and the findings?	yes	4. Discussion
31.	Clarity of major themes	Were major themes clearly presented in the findings?	yes; subheadings according to themes; figures	3.2 Qualitative content analyses
32.	Clarity of minor themes	Is there a description of diverse cases or discussion of minor themes?	yes	3.2 Qualitative content analyses

From: Tong, Allison; Sainsbury, Peter; Craig, Jonathan (2007): Consolidated criteria for reporting qualitative research (COREQ): a 32-item checklist for interviews and focus groups. In: International journal for quality in health care: journal of the International Society for Quality in Health Care 19 (6), S. 349–357. DOI: 10.1093/intqhc/mzm042
